# Excited-State Barrier Controls *E* → *Z* Photoisomerization in *p*-Hydroxycinnamate Biochromophores

**DOI:** 10.1021/acs.jpclett.2c02613

**Published:** 2022-09-23

**Authors:** Eleanor K. Ashworth, Neville J. A. Coughlan, W. Scott Hopkins, Evan J. Bieske, James N. Bull

**Affiliations:** † School of Chemistry, University of East Anglia, Norwich NR4 7TJ, United Kingdom; ‡ Department of Chemistry, University of Waterloo, Waterloo, Ontario N2L 3G1, Canada; § WaterMine Innovation, Inc., Waterloo, Ontario N0B 2T0, Canada; ∥ School of Chemistry, University of Melbourne, Parkville, VIC 3010, Australia

## Abstract

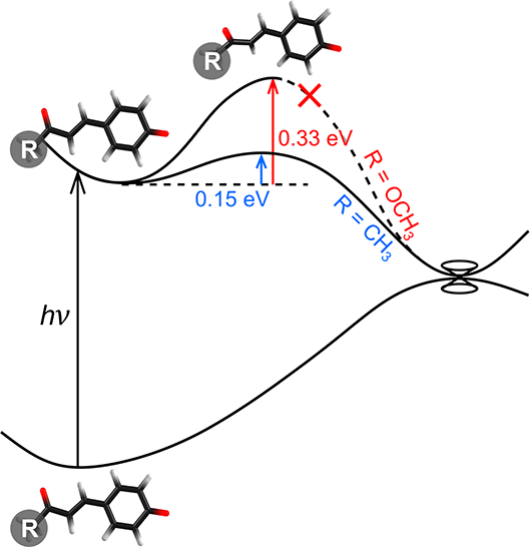

Molecules based on the deprotonated *p*-hydroxycinnamate moiety are widespread in nature, including serving as UV filters in the leaves of plants and as the biochromophore in photoactive yellow protein. The photophysical behavior of these chromophores is centered around a rapid *E* → *Z* photoisomerization by passage through a conical intersection seam. Here, we use photoisomerization and photodissociation action spectroscopies with deprotonated 4-hydroxybenzal acetone (*p*CK^–^) to characterize a wavelength-dependent bifurcation between electron autodetachment (spontaneous ejection of an electron from the S_1_ state because it is situated in the detachment continuum) and *E* → *Z* photoisomerization. While autodetachment occurs across the entire S_1_(ππ*) band (370–480 nm), *E* → *Z* photoisomerization occurs only over a blue portion of the band (370–430 nm). No *E* → *Z* photoisomerization is observed when the ketone functional group in *p*CK^–^ is replaced with an ester or carboxylic acid. The wavelength-dependent bifurcation is consistent with potential energy surface calculations showing that a barrier separates the Franck–Condon region from the *E* → *Z* isomerizing conical intersection. The barrier height, which is substantially higher in the gas phase than in solution, depends on the functional group and governs whether *E* → *Z* photoisomerization occurs more rapidly than autodetachment.

Molecules possessing the *p*-hydroxycinnamate moiety are widespread in nature.^1^ Examples include sinapoyl malate, caffeic acid, and ferulic acid in both the free form and covalently bound to cell walls and lignin structures, which are present in the leaves, stems, and seeds of plants where they function as UV-B filters.^2^ The UV-B filtering mechanism is, in part, thought to rely on a rapid internal conversion to the ground state, accompanied by *E* → *Z* photoisomerization.^3^ The efficacy of this nonradiative decay has prompted the skin-care industry to develop commercial sunscreens containing cinnamate-based molecules.^4,5^ In another biological context, deprotonated *p*-hydroxycinnamates are invoked as models for the chromophore in photoactive yellow protein (PYP), which is a small blue-light sensing protein found in the *Halorhodospira halophila* bacterium.^6−8^ In the PYP photocycle, absorption of blue light by a thioester-based hydroxycinnamate chromophore leads to an *E* → *Z* photoisomerization of the chromophore, which in turn leads to a change in protein conformation and eventually a negative phototaxis response of the bacterium.^9−12^

A desire to understand the photophysics of *p*-hydroxycinnamates and to develop synthetic derivatives that might be incorporated into optogenetic applications^13−15^ or skin-care products^16^ has prompted numerous investigations on the inherent photophysics in this class of molecules. Although the excited-state dynamics in *p*-hydroxycinnamates has been studied extensively in solution over the past two decades (see, for example, refs (17−22) and references therein), the extent to which solvation perturbs the intrinsic excited-state dynamics remains unclear. Theoretical investigations have suggested that solvation of anionic *p*-hydroxycinnamates significantly perturbs the S_1_(ππ*) potential energy surfaces and conical intersection seams,^23−29^ although experimental strategies capable of directly observing photoisomerization in the gas phase are now starting to emerge.^30−32^

Previous experiments on hydroxycinnamate anions, focusing on the inherent dynamics, utilized techniques including time-resolved photoelectron spectroscopy,^33−35^ frequency-resolved photoelectron spectroscopy to fingerprint internal conversion dynamics,^36^ and photoisomerization action (PISA) spectroscopy to select precursor deprotomers or geometric isomers and to probe photoisomerization or phototautomerization.^32,37^ However, these studies were unable to provide any evidence for *E* → *Z* photoisomerization across a series of around 20 hydroxycinnamate anions. The present study provides clear evidence for an *E* → *Z* photoisomerization response in deprotonated 4-hydroxybenzal acetone (*p*CK^–^, Figure 1a), a molecule which has been invoked as a proxy for the PYP chromophore.^18,24,26,33,35,38^ Importantly, the *E* → *Z* photoisomerization response occurs only following excitation of the higher photon energy region of the S_1_ ← S_0_ absorption band, consistent with earlier molecular dynamics simulations hypothesizing that photoisomerization by passage through a conical intersection is a barrier-controlled process.^39−41^ Our study provides experimental confirmation that functional group substitution on the hydroxycinnamate tail critically affects the excited-state barrier height and thus photoisomerization efficacy.^24,26,42^ Comparison of time-resolved data for *p*CK^–^ in solution with gas phase data implies that the potential energy surface barrier to isomerization is stabilized in solution.

**Figure 1:**
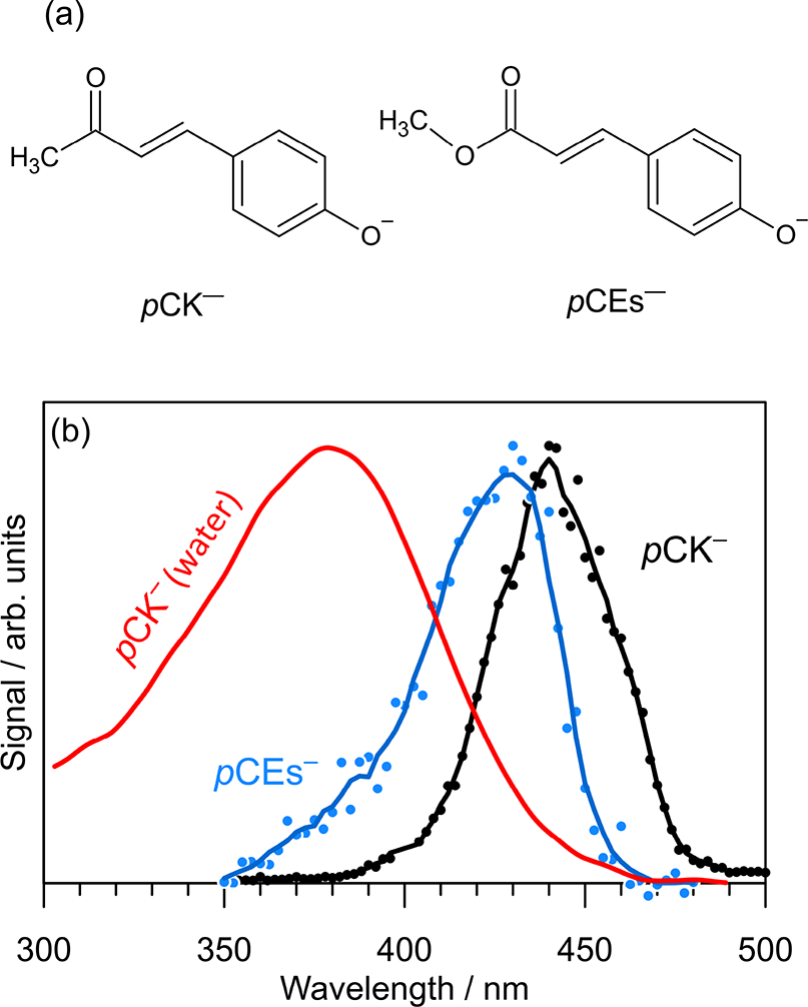
(a) *p*-Hydroxycinnamate anions considered in this study. (b) Action spectra for *p*CK^–^ (photodissociation, black) and *p*CEs^–^ (photodetachment, blue, from ref (32)) as proxies for the S_1_ ← S_0_ absorption bands. The absorption spectrum of *p*CK^–^ in water (at *T* = 300 K) is shown in red. Solid lines are moving averages over three data points for the gas phase and seven data points for the condensed phase data.

A photodissociation action spectrum (black), which serves as a proxy for the visible absorption spectrum of *p*CK^–^, is shown in Figure 1b. The spectrum was recorded by monitoring absorption-induced fragmentation of the anion under ultrahigh-vacuum conditions (see the Supporting Information for experimental details). The spectrum spans the 390–480 nm range with maximum response at 440 nm. The spectrum is red-shifted by ≈10 nm compared with the photodetachment action spectrum for the methyl ester (*p*CEs^–^) from ref (32) (see also ref (43)), corresponding to absorption-induced electron ejection. The origin of the red-shift for *p*CK^–^ is presumably due to differences in inductive electron donation. The absorption spectrum of pCK- in water is blue-shifted by ≈80 nm relative to the photodissociation spectrum.

Photoisomerization of isolated *p*CK^–^ was investigated using the emerging technique of PISA spectroscopy. A detailed description and illustration of the PISA spectroscopy technique are available in refs (30 and 44). Briefly, PISA spectroscopy allows for isomer-selected irradiation experiments, isomer-specific product detection, and quantification of photodetached electrons for anions using an electron scavenger (SF_6_).^32,44^ In an experiment, charged isomers that are drifting under the influence of an electric field through a buffer gas (e.g., N_2_ or CO_2_) are separated according to their drift speeds, which depend on their collision cross sections. The target isomer is selected in a primary drift stage and then exposed to wavelength tunable light, with separation of photoisomers or photofragments in a second drift stage. By monitoring the yield of photoisomers and  as a function of wavelength, photoisomerization and photodetachment action spectra are recorded. Complete experimental details are given in the Supporting Information.

Electrospray ionization of *p*CK^–^ in any of the buffer gases considered in this study produced a single arrival time distribution (ATD) peak, consistent with a single isomer (black traces in Figure 2a,b) assigned to the *E* configuration. In pure N_2_ buffer gas, the photoaction ATD in Figure 2a, corresponding to the difference between “light on” and “light off” ATDs, shows generation of a photoisomer at a slightly shorter arrival time, consistent with the *Z* isomer, since cross-section modeling predicts that the *Z* isomer has a smaller collision cross section in pure N_2_ (Table 1). Similar results were obtained in pure CO_2_ buffer gas (see the Supporting Information). The photoaction ATD in N_2_ buffer gas doped with ≈1% SF_6_ and ≈1% propan-2-ol shows generation of a photoisomer at longer arrival time (assigned to *Z*) and electron detachment as detected through  formation when using 420 nm light. Further explanation on isomer-specific interactions with propan-2-ol leading to the increased collision cross section for the *Z* isomer compared with the *E* isomer is given in the Supporting Information. Because the photodepletion signal (i.e., bleach of the *E* isomer) in Figure 2b is balanced by the sum of photoisomerization and electron detachment signals, the experiment captures all prompt photoaction. This correspondence is true across all wavelengths considered in this study. Notably, there is no photodissociation in this experiment because collisional energy quenching (tens to hundreds of nanoseconds) occurs more rapidly than recovery of the ground electronic state followed by statistical dissociation (microseconds).^32,45^

**Table 1:** Calculated Properties for the *E* and *Z* Isomers of *p*CK^–^

species	Δ*E*^a^	ADE^a^	VDE^a^	Ω_c_
(*E*)-*p*CK^–^	0	2.83	2.90	137
(*Z*)-*p*CK^–^	27	2.80	2.86	136
TS^b^	125			
expt		2.8 ± 0.1^c^	3.0 ± 0.1^c^	134 ± 5^d^

a Δ*E* in units of kJ mol^–1^; ADE (adiabatic detachment energy) and VDE (vertical detachment energy) in units of eV.

b TS is the isomerization transition state on the ground electronic state. All energies at the DLPNO-CCSD(T)/aug-cc-pVTZ level of theory using ORCA 5.0.3.^46^ Ω_c_ in units of Å^2^, calculated using MOBCAL.^47,48^

c Reference (35).

d (*E*)-*p*CK^–^.

**Figure 2:**
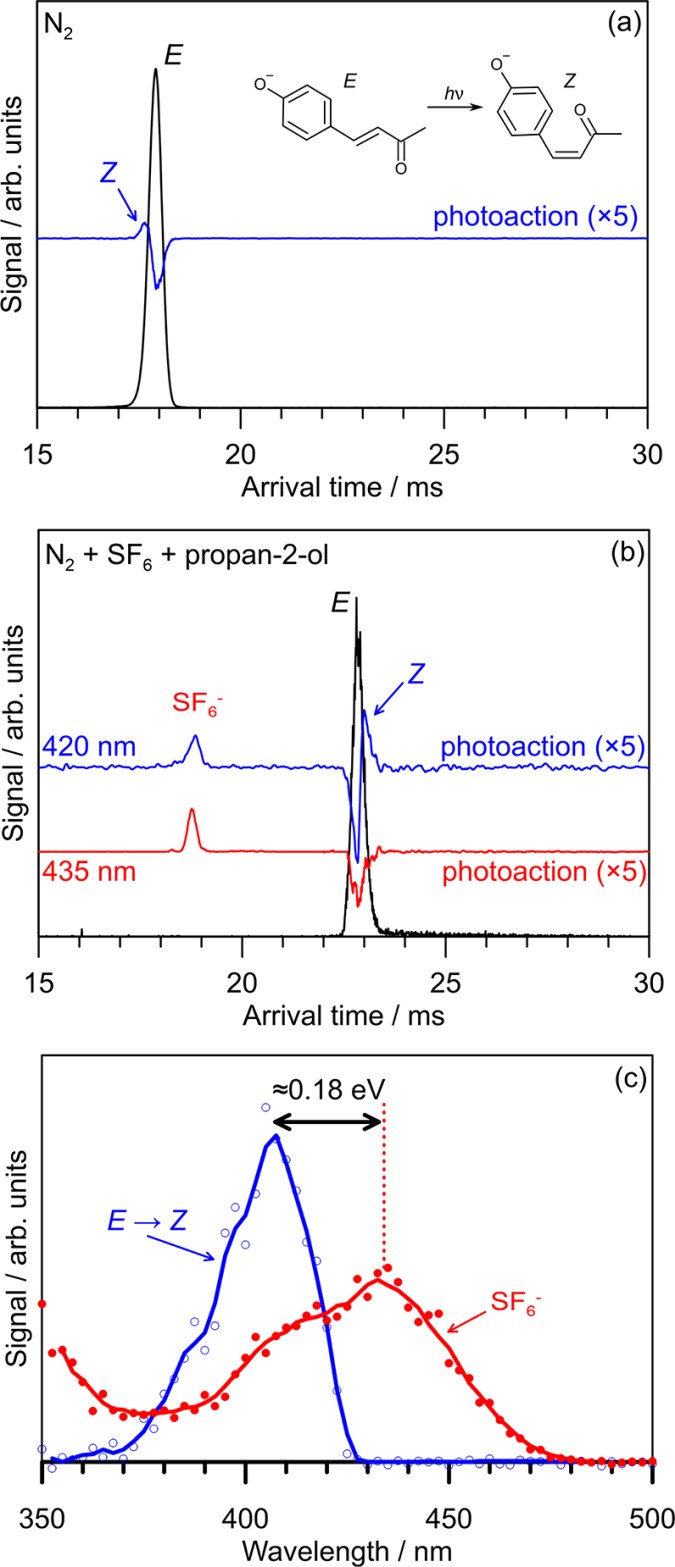
Action spectroscopy of *p*CK^–^: (a) light-off (black) and photoaction (blue) ATD at 420 nm in pure N_2_ buffer gas; (b) light-off (black) and photoaction (blue, 420 nm and red, 435 nm) ATD in N_2_ buffer gas seeded with ≈1% propan-2-ol and ≈1% SF_6_; (c) electron photodetachment (red) and *E* → *Z* photoisomerization (blue) action spectra. The photoaction spectra show the changes between light-on and light-off ATDs, reflecting any photoinduced processes. The photoisomerization quantum yield is estimated at a 1–2% at 400 nm. See the Supporting Information for CO_2_ buffer gas data. The excited-state barrier to isomerization is estimated at ≈0.18 eV from the difference in spectral maxima in (c); use of thresholds is not reliable because of hot bands and the direct photodetachment contribution to electron detachment because the S_1_ state is situated in the detachment threshold.

Photodetachment (red) and photoisomerization (blue) action spectra for *p*CK^–^ are shown in Figure 2c. While electron detachment is observed across the 370–480 nm range with maximum response at ≈435 nm, *E – Z* isomerization was observed only over the 360–430 nm range with maximum response at ≈405 nm. These spectra span the same wavelength range as the photodissociation spectrum (proxy for the absorption spectrum) in Figure 1b, although differing in shape, which is indicative of competitive photochemical pathways. It is worth noting that electron detachment may occur following absorption of a single photon because the onset of the action spectra is situated above the adiabatic detachment energy (Table 1) when allowing for the internal energy associated with temperature of the ions at *T* = 300 K (0.3 eV). This situation is also true for *p*CEs^–^.^32,34,36,37^

In PISA spectroscopy, an isomerization signal can result from two mechanisms: (i) a rapid excited-state process associated with passage through a conical intersection and (ii) statistical isomerization on the ground electronic state before collisions in the drift region thermalize the activated ions. In an earlier study considering *p*CEs^–^,^32^ we used master equation simulations combining RRKM isomerization rates with Langevin collisional energy quenching to explore the possibility of process ii, where it was concluded to be unlikely because of the electronic energy difference between the two isomers, although some *Z* → *E* thermal reversion may occur before collisions stabilize the isomers. To investigate thermal reversion (process ii) for *p*CK^–^, which may skew the appearance of the photoisomerization action spectra, we considered an experimental approach in which the ion mobility experiments were repeated in CO_2_ buffer gas (see the Supporting Information). The rationale is that the vibrational energy quenching collision cross section for CO_2_ is an order of magnitude larger than that for N_2_,^49^ providing more rapid thermalization and suppression of ground-state statistical processes. Because the action spectra in CO_2_ buffer gas closely resemble those shown in Figure 2c, it is unlikely that *Z* → *E* thermal reversion processes have a significant bearing on the action spectra.

The occurrence of *E* → *Z* photoisomerization for *p*CK^–^ in the gas phase contrasts with *p*CEs^–^ and derivatives such as the phenoxide deprotomer of *p*-coumaric acid and ring-substituted derivatives caffeic, ferulic, and sinapinic acid (and methyl esters of each), for which no *E* → *Z* isomerization was observed.^32,37^ Earlier molecular dynamics simulations and related studies have suggested that there is a barrier on the S_1_ state potential energy surface for double-bond rotation (β-torsion coordinate in Figure 3a).^39−41^ A recent study of *p*CK^–^ calculated a barrier of ≈0.25 eV along the β-torsion coordinate,^35^ although this value is based on linear interpolation of internal coordinates between the Franck–Condon geometry and the double-bond twisted minimum-energy structure. This value is lower than the earlier calculated barrier of ≈0.4 eV, relative to the Franck–Condon geometry, found for *p*CEs^–^ using the DLPNO-STEOM-CCSD/aug-cc-pVDZ method.^34^ To enable a robust comparison between *p*CK^–^ and *p*CEs^–^, we optimized the β-torsion critical points along the S_1_ potential energy surfaces (Figure 3) using a CASSCF(10,9) wave function followed by XMCQDPT2 energy calculations (Table 2) using the Firefly 8.2.0 software package.^50^ These calculations gave a barrier of 0.15 eV for *p*CK^–^ and 0.33 eV for *p*CEs^–^, with torsion of the β coordinate of 53.5° (*p*CK^–^, −238 cm^–1^) and 49.1° (*p*CEs^–^, −325 cm^–1^) at the transition state. Significantly, the computed barrier for *p*CK^–^ is in good agreement with experiment (≈0.18 eV) and is substantially lower than that for *p*CEs^–^.

**Table 2:** Calculated Potential Energy Surface Critical Points in eV at the XMCQDPT2(10,9)/aug-cc-PVDZ Level of Theory for *p*CK^–^ and *p*CEs^–^

	*p*CK^–^	*p*CEs^–^
VEE^a^	2.87	2.96
S_1_ min	2.69	2.85
S_1_ TS^‡^^b^	0.15	0.33
*E–Z* CI	2.62	2.82

a VEE = vertical excitation energy.

b Relative to S_1_ min. Values relative to the Franck–Condon geometry are given in the text.

**Figure 3:**
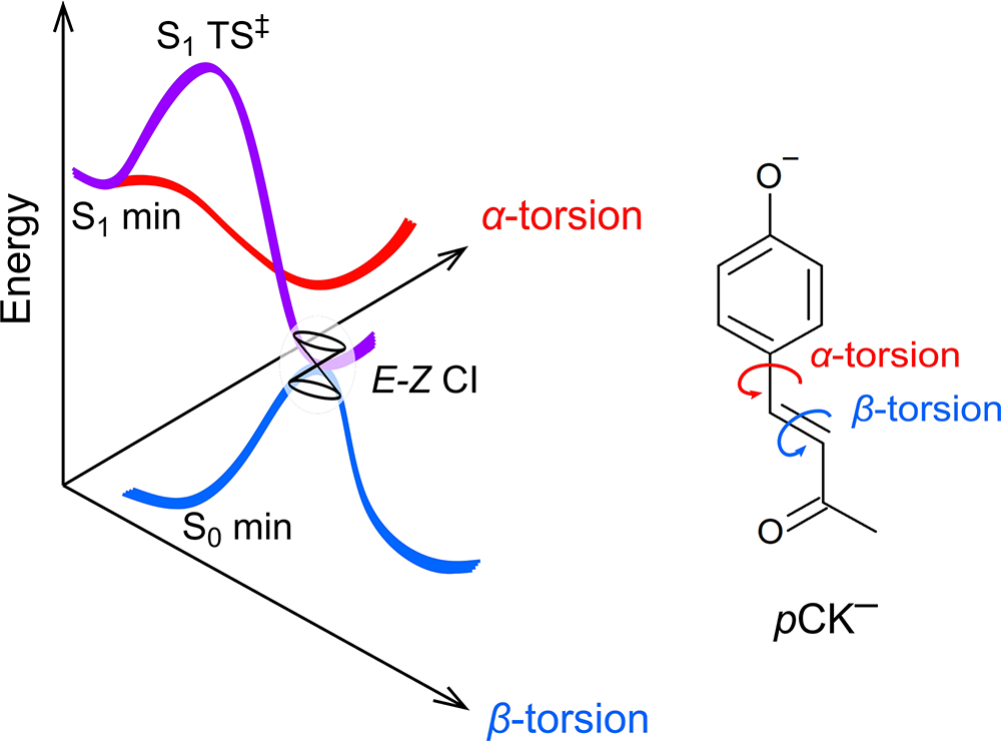
Schematic illustration of potential energy surfaces for the *E* isomer of *p*CK^–^ showing the α and β coordinates and identifying S_0_ and S_1_ minimum-energy geometries, the β-coordinate transition state (S_1_ TS^‡^), and the *E–Z* minimum-energy conical intersection (CI). Calculated energies for these critical points are given in Table 2. The α coordinate has been considered in ref (35).

Having established the intrinsic *E* → *Z* photoisomerization response for *p*CK^–^ at *T* = 300 K, we next considered excited-state lifetimes. Following rapid geometric relaxation from the Franck–Condon geometry (τ_1_ < 1 ps), gas phase lifetimes for *p*CK^–^ have been determined to be τ_2_ = 52 ps when pumped at 400 nm^33^ and ≈120 ps when pumped at 444 nm,^35^ with the latter corresponding to exciting near the absorption band maximum. While the 400 nm study observed ground-state recovery (and assumed isomerization), the 444 nm study observed only autodetachment (spontaneous ejection of an electron from the S_1_ state because it is situated in the detachment continuum) and predicted α-torsion dynamics based on photoelectron angular distributions. The present action spectra shown in Figure 2c confirm that both time-resolved studies reached correct conclusions: excitation at 400 nm leads to isomerization while excitation at 444 nm does not. These dynamics contrast with *p*CEs^–^, which, when pumped near the maximum in its absorption band (438 nm), decays exclusively thorough autodetachment with a lifetime of 45 ± 4 ps.^34^ Similar autodetachment processes have been fingerprinted over the entire absorption band for *p*CEs^–^ with no evidence for internal conversion and thus the possibility of *Z* isomer formation.^36^ The longer excited-state lifetime for *p*CK^–^ compared with *p*CEs^–^ when pumped near the maximum in the photodissociation action spectrum is presumably because, while the two anions have similar electron detachment thresholds, the absorption profile for *p*CK^–^ is red-shifted by ≈0.1 eV (Figure 1), decreasing the propensity for electron autodetachment following rapid nuclear relaxation away from the Franck–Condon geometry.

To facilitate a comparison of gas phase excited-state lifetimes with those in solution, we performed time-resolved fluorescence upconversion (≈50 fs time resolution) on *p*CK^–^ dissolved in a series of polar solvents at *T* = 300 K.^51,52^ Fluorescence upconversion is a time-resolved spectroscopic technique in which the fluorescence emission from a sample is frequency mixed with a probe laser pulse (800 nm), producing an “upconverted” signal. By changing the delay between femtosecond pump and probe pulses, and monitoring the upconverted signal, fluorescence lifetimes are measured. The present upconversion measurements refine an earlier solvent polarity and viscosity upconversion study on *p*CK^–^ ^18^ and were performed because the earlier study (i) was limited by ≈500 fs time resolution, (ii) used an excitation wavelength (340 nm), which is far from the absorption maximum and likely accesses a nπ* state that gains substantial intensity through Herzberg–Teller coupling,^36^ and (iii) assumed static samples that likely gave rise to photostationary states. Steady-state fluorescence excitation and emission spectra for *p*CK^–^ in water are shown in Figure 4a, revealing a large Stokes shift of 5794 ± 20 cm^–1^ (4143 ± 20 cm^–1^ in ethanol; see further data in the Supporting Information); the large shift is in part attributed to hydrogen-bond interactions between the phenoxide group and solvent molecules weakening upon excitation.^17,19,41^ The Stokes shift at *T* = 77 K is significantly lower at 2243 ± 20 cm^–1^ (ethanol), consistent with inhibition of nuclear and/or solvent relaxation to reach the lowest energy fluorescing geometry.

**Figure 4:**
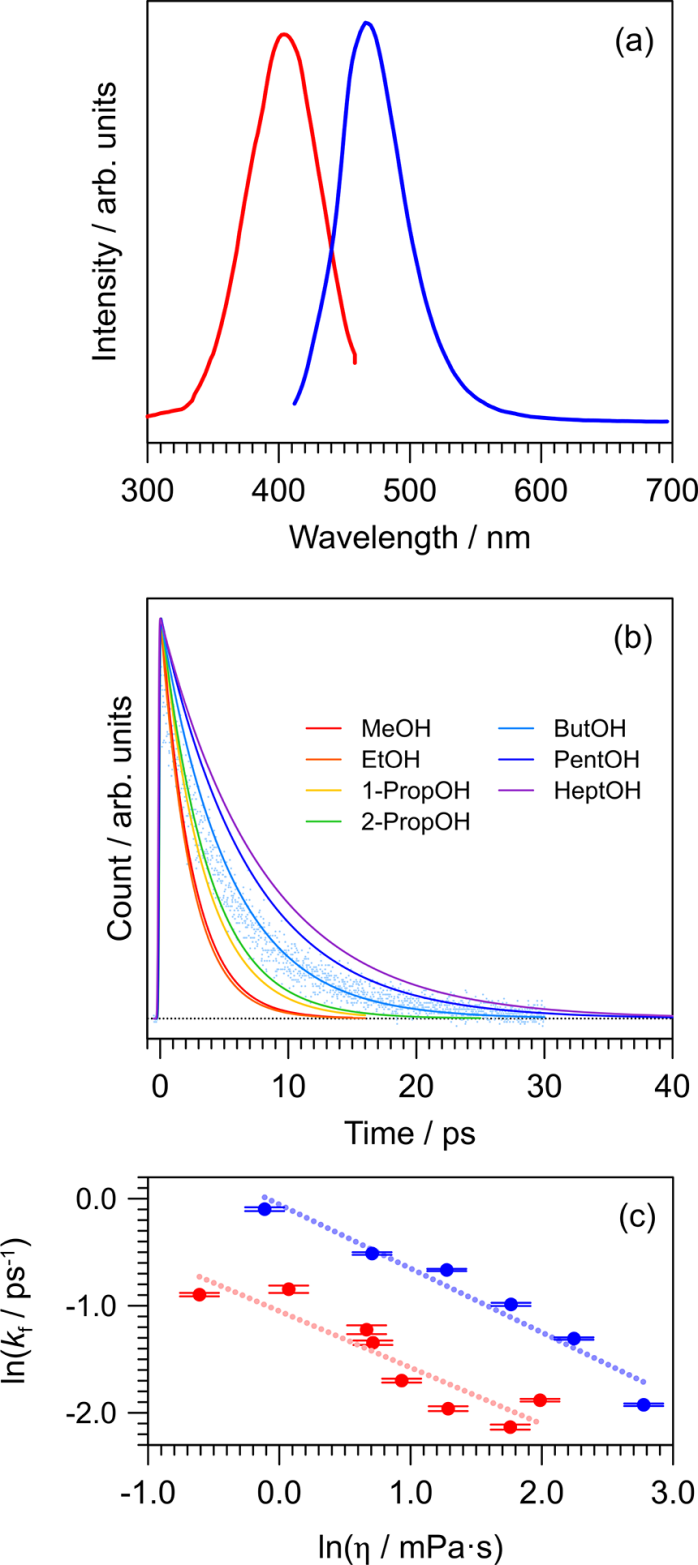
Fluorescence spectroscopy of *p*CK^–^ in solution at *T* = 300 K: (a) excitation (red, monitoring at 480 nm) and emission (blue, exciting at 400 nm) fluorescence spectra in water; (b) time-resolved fluorescence upconversion decay curves and model fits in a series of alcohols (fitted values are given in the Supporting Information). Experimental data points are shown for ButOH; (c) viscosity (η) effect of  in a series of alcohols (red) and water–ethylene glycol mixtures (blue).

Fluorescence upconversion data following excitation of *p*CK^–^ with 400 nm light, which were measured in a flow cell at *T* = 300 K, are shown in Figure 4b; results for water–ethylene-glycol mixtures are given in the Supporting Information. The decay curves were fit with a two-component exponential decay model with lifetime τ_1_ dominated by rapid solvent rearrangement (limited by the cross correlation), and τ_2_ is linked to the excited-state lifetime and associated solvent motion, i.e., convoluted with the ≈880 fs longest time scale dynamics for water rearrangement.^53^ Fitted lifetimes in selected solvents are given in Table 3 (see all data in the Supporting Information). It is worth nothing that the ≈1 ps lifetime of *p*CK^–^ in water is comparable with the time scale for *E* → *Z* photoisomerization of the chromophore in PYP.^11,54^ It is striking that the excited-state lifetimes for *p*CK^–^ in solution are 1 or 2 orders of magnitude shorter than in the gas phase, suggesting a considerable reduction of the isomerization barrier in solution or access of an alternative relaxation pathway. This situation contrasts with anionic retinoids in the gas phase that undergo barrier-controlled stereospecific *E*–*Z* photoisomerization and have considerably shorter lifetimes than in solution.^55^ Studies of derivative hydroxycinnamate chromophores in solution have shown that the excited-state lifetime is sensitive to the identity of the functional group on the carbonyl tail,^19^ with the ketone group for *p*CK^–^ giving rise to the shortest lifetimes, although an overall picture is complicated because of solvent polarity effects, differences in charge-transfer character, and hydrogen bonding.^20^

**Table 3:** Fitted Excited-State Lifetimes (τ_2_ in ps) for *p*CK^–^ in Water and Alcohol Solvents at *T* = 300 K

species	τ_2_	±
water	1.17	0.01
MeOH	2.45	0.02
EtOH	2.33	0.04
1-PropOH	3.40	0.07
2-PropOH	3.84	0.04
ButOH	5.47	0.05
PentOH	7.11	0.08
HeptOH	8.44	0.10
OctOH	6.57	0.04

The influence of viscosity on the excited-state lifetime of *p*CK^–^ is shown in Figure 4c revealing a strong effect, consistent with an isomerization-type reaction. See the Supporting Information for the solvent polarity effect. Following ref (18), excited-state lifetimes as a function of viscosity were fit with the phenomenological power law ,^56^ where *k*_f_ is assumed as the photoisomerization rate and *C* is proportional to the Arrhenius term  and is linked to polarity dependence (stabilization) of the transition state. The parameter α is a measure of the viscosity effect for isomerization, which approaches unity in highly viscous solvents.^57^ Fitted values of α are 0.53 and 0.59 for the alcohols and water–ethylene glycol mixtures, respectively, with the latter being slightly larger than that reported in ref (18). Assuming an Arrhenius relation at *T* = 300 K and a pre-exponential factor of  s^–1^, the excited-state barrier height in water is ≈0.07 eV, which is around half of the gas phase value. This estimate is consistent with potential energy surface calculations on the dianion of *p*-coumaric acid with microhydration, showing a reduction of the barrier from 0.70 eV (gas phase) to 0.09 eV. Values of α have been determined at 0.64 for *p*CEs^–^ ^18^ and 0.75 for the thioester anion,^17^ consistent with *p*CK^–^ having the lowest isomerization barrier in solution. We conclude that solvation significantly stabilizes the barrier to isomerization.

In summary, this study has demonstrated that *E* → *Z* photoisomerization of a *p*-hydroxycinnamate anion may occur in the gas phase, although a barrier to double-bond torsion on the S_1_(ππ*) potential energy surface is a key factor in defining if photoisomerization is competitive with electron autodetachment. Substitution on the carbonyl group tunes the barrier height separating the Franck–Condon geometry and the *E–Z* isomerizing conical intersection seam. Solvation of the chromophore significantly stabilizes the excited-state barrier, leading to rapid nonradiative relaxation. The present experimental strategy is applicable to other charged systems that may photoisomerize and possess excited-state barriers, and is particularly applicable to systems for which there are wavelength-dependent dynamics leading to multiple isomeric products.^55^ Future work on deprotonated hydroxycinnamate anions will seek to photogenerate, isolate, and apply frequency and time-resolved action spectroscopy techniques to *Z* isomers, as well as other unstable or intermediate isomers such as *keto–enol* tautomers,^32^ to map out excited-state potential energy surfaces.

## Supporting Information Available

Experimental methods; theoretical methods; CASSCF orbitals; vertical excitation energies; action spectroscopy in CO_2_ buffer gas; *p*CK^–^·propan-2-ol complexes; solution spectroscopy; critical point geometries (PDF)
