# Supplementary material for: Excited-State Barrier Controls E → Z Photoisomerization in p-Hydroxycinnamate Biochromophores
Source: J Phys Chem Lett. 2022 Sep 23;13(39):9028–34. doi: 10.1021/acs.jpclett.2c02613 (PMC9549896; doi:10.1021/acs.jpclett.2c02613)
Supplement: Supplementary file 1 — jz2c02613_si_001.pdf [file jz2c02613_si_001.pdf]

Supporting Information for 'Excited-State Barrier Controls $E \rightarrow Z$ Photoisomerization in *para*-hydroxycinnamate Biochromophores'

Eleanor K. Ashworth,[†] Neville J. A. Coughlan,[‡,§] W. Scott Hopkins,[‡,§] Evan J. Bieske,[¶] and James N. Bull[*,†]

†*School of Chemistry, University of East Anglia, Norwich NR4 7TJ, United Kingdom*
‡*Department of Chemistry, University of Waterloo, Waterloo N2L 3G1, Canada*
¶*School of Chemistry, University of Melbourne, Parkville, VIC 3010, Australia*
§*WaterMine Innovation, Inc., Waterloo, Ontario N0B 2T0, Canada*

E-mail: james.bull@uea.ac.uk

Experimental methods

Photodissociation action spectroscopy

The photodissociation action spectrum for pCK$^-$ was recorded in a modified DMS-MS device capable of irradiating target anions in the third quadrupole region of a triple quadrupole mass spectrometer with tunable wavelength laser light. Complete details of the DMS-MS apparatus are given in Refs 1–3; for a description of the instrument modifications enabling photodissociation action spectroscopy see Ref. 4. The target anion was electrosprayed from acetonitrile ($\approx$1 μg mL^{-1}) with 0.1% NH_4OH (to assist deprotonation). Ions were carried through the planar DMS cell by dry nitrogen gas. Ionograms showed evidence for only one isomer, consistent with the tandem ion mobility experiments detailed in the next section. For the photodissociation measurements, the compensation voltage applied to the DMS cell was fixed to transmit ions from individual ion populations into a triple-quadrupole mass spectrometer (Q1–Q3). Ions were mass selected in Q1 (m/z 161), transmitted through Q2 and accumulated in Q3 for $\approx$10 ms. Trapped ions were irradiated for $\approx$500 ms with light from a tunable optical parametric oscillator (OPO, Horizon II, Continuum, loosely focused, 2–5 mJ pulse^{-1}) pumped by a pulsed Nd:YAG laser (10 Hz, Surelite, Continuum). The parent ions and any photofragment ions were ejected from the ion trap following a mass-selective axial ejection toward a channeltron ion detector.[5] Ion fragmentation efficiencies were normalized with respect to ion count, OPO fluence, and photon number. Photodissociation was dominated by two fragments, corresponding to loss of CH_3 and C_2H_4O.

Photoisomerizaiton action spectroscopy

Photoisomerization and prompt photodetachment of pCK$^-$ was investigated in a custom tandem ion mobility spectrometer (IMS-IMS) coupled with a quadrupole mass filter (QMF).[6,7] Briefly, pCK$^-$ was produced through electrospray ionisation of a $\approx$10 μmol L^{-1} solution (Sigma-Aldrich, >99 %) dissolved in methanol (voltage -3 kV, flow rate $\approx$10 μL min^{-1}). Electrosprayed ions were transferred *via* a heated capillary into a RF ion funnel (IF1), which radially gathered and confined the ions. An ion gate (IG1) at the end of IF1 injected $\approx$100 μs packets of ions at 40 Hz into the first IMS drift region (IMS1) where they were propelled by an electric field (44 V cm^{-1}) through pure N_2 or CO_2 buffer gas, N_2 seeded with $\approx$1% SF_6 and $\approx$1% propan-2-ol, or CO_2 seeded with $\approx$1% propan-2-ol (see Supporting Information) at a pressure of $\approx$6 Torr. The isomers become separated spatially and temporally because more extended ions (E isomers) have different collision cross-sections with the buffer gas than the Z isomers.[8] After traversing the drift regions (IMS1 + IMS2), a second ion funnel collected the ions and introduced them into a differentially pumped octupole ion guide and quadrupole mass filter that mass-selected the ions before they reached the ion detector. The detector was connected to a multichannel scaler that produced a histogram of ion counts against arrival time, t, corresponding to an arrival time distribution (ATD). In all presented ATDs, t=0 corresponds to the opening of IG1. The mobility resolution, $t/\Delta t$, for singly-charged anions is typically 80 – 90.[9]

For the action spectroscopy measurements, packets of ions with similar collision cross-sections were selected using a Bradbury-Nielsen ion gate after IMS1 (IG2, $\approx$100 μs opening time). Immediately after gating, the mobility-selected ions were excited with a pulse of light from an optical parametric oscillator (OPO, EKSPLA NT342B). Any change in the ions' collision cross-section due to photoisomerization was manifested as a shift in arrival time following passage through a second IMS drift region (IMS2). The OPO was operated at 20 Hz, half the rate of ion injection, allowing accumulation of light-on and light-off ATDs. The difference between the light-on and light-off ATDs reflected the photoresponse (photoaction ATD). Action spectra were derived by integrating

the photoaction ATD signal, and normalizing with respect to light pulse fluence and total laser-off signal at each wavelength. The action spectroscopy measurements were performed with a light pulse fluence of $< 0.5\,\mathrm{mJ\,cm^{-2}}$ to minimize multiphoton absorption and sequential photoisomerizations.

Time-resolved fluorescence upconversion

Time-resolved fluorescence upconversion measurements were performed using an instrument that has been previously detailed.[10] Briefly, a CW Nd:YVO4 laser drives a Kerr lens mode-locked Ti:Sapphire (Ti:S) oscillator generating ≈800 nm light in ≈20 fs pulses at 80 MHz. The second harmonic (400 nm, ≈11 mW) was generated by focussing the fundamental (≈840 mW) light into a 50 μm thickness barium borate crystal (BBO, type I) using a 150 mm focal length concave mirror. The fundamental and second harmonic wavelengths were separated with a dichroic mirror. The 400 nm and 800 nm light pulses were delayed relative to each other using a computer-controlled motorised delay stage (0.1 μm resolution). The use of chirped mirrors minimized temporal broadening. The 400 nm pump pulse was focussed onto the sample held in either a static 2 mm path length quartz cell or 2 mm path length quartz flow cell using a concave mirror. Resulting fluorescence was captured and focussed by a microscope objective (15x magnification) through a CG455 Schott filter into a 100 μm thickness BBO crystal (type I) and frequency mixed with 800 nm light. The unconverted signal was passed through a UG11 Schott filter into a monochromator (Photon Technology International Model 101, resolution 2 nm/mm) and low-noise photomultiplier (PMT, Hamamatsu R585). PMT signal is connected to a computer-interfaced Stanford Research Systems photon counter (SR400). The cross correlation of the instrument has been characterised through Raman scattering in heptane to be ≈55 fs.

The fluorescence quantum yield for $p\mathrm{CK}^-$ at $T = 300\,\mathrm{K}$ has been estimated at less than 10^{-3}.[11] The flow rate in the flow cell was systematically increased until measured up-converted lifetimes no longer changed with increasing flow rate.

Theoretical methods

Electronic structure calculations

Electronic structure calculations were performed using the Gaussian 16.B01, Firefly 8.2.0, and ORCA 5.0.3 software packages.[12–14] Geometrical optimizations, vibrational frequencies, and isomerization transition state searches were performed at the ωB97X-D/aug-cc-pVTZ level of theory,[15,16] followed by single-point energy calculations at the DLPNO-CCSD(T)/aug-cc-pVTZ level of theory.[17] Vertical detachment energies (VDEs) were calculated at the EOM-IP-CCSD/aug-cc-pVDZ level of theory, based on good performance in a recent study.[9]

Collision cross-sections were calculated using MOBCAL with the trajectory method parametrised for N_2 buffer gas.[18,19] Input charge distributions were computed at the ωB97X-D/aug-cc-pVDZ level of theory with the Merz-Singh-Kollman scheme constrained to reproduce the electric dipole moment.[20] Sufficient trajectories were computed to give standard deviations of ± 1 Å^2 for the calculated values.

For the potential energy surface calculations, S_0 and S_1 state equilibrium geometries, TS‡, and conical intersection geometries were optimized at the state-specific CASSCF(10,9)/aug-cc-pVDZ level of theory. Single-point energy calculations were performed at the XMCQDPT2(12,11)/aug-cc-pVDZ level of theory. The active spaces chosen for the geometry optimizations and single-point energy calculations are a balance between accuracy and computation cost following the earlier study by Boggio-Pasqua and Groenhof.[21]

CASSCF orbitals

Optimized CASSCF orbitals using a (10,9) active space for (*E*)-*p*CK$^-$ and (*E*)-*p*CEs$^-$ are shown in Figures S1 and S2, respectively. In both cases, the $S_1 \leftarrow S_0$ transition has $\pi - \pi^*$ character with the dominant configuration involving a HOMO-LUMO transition.

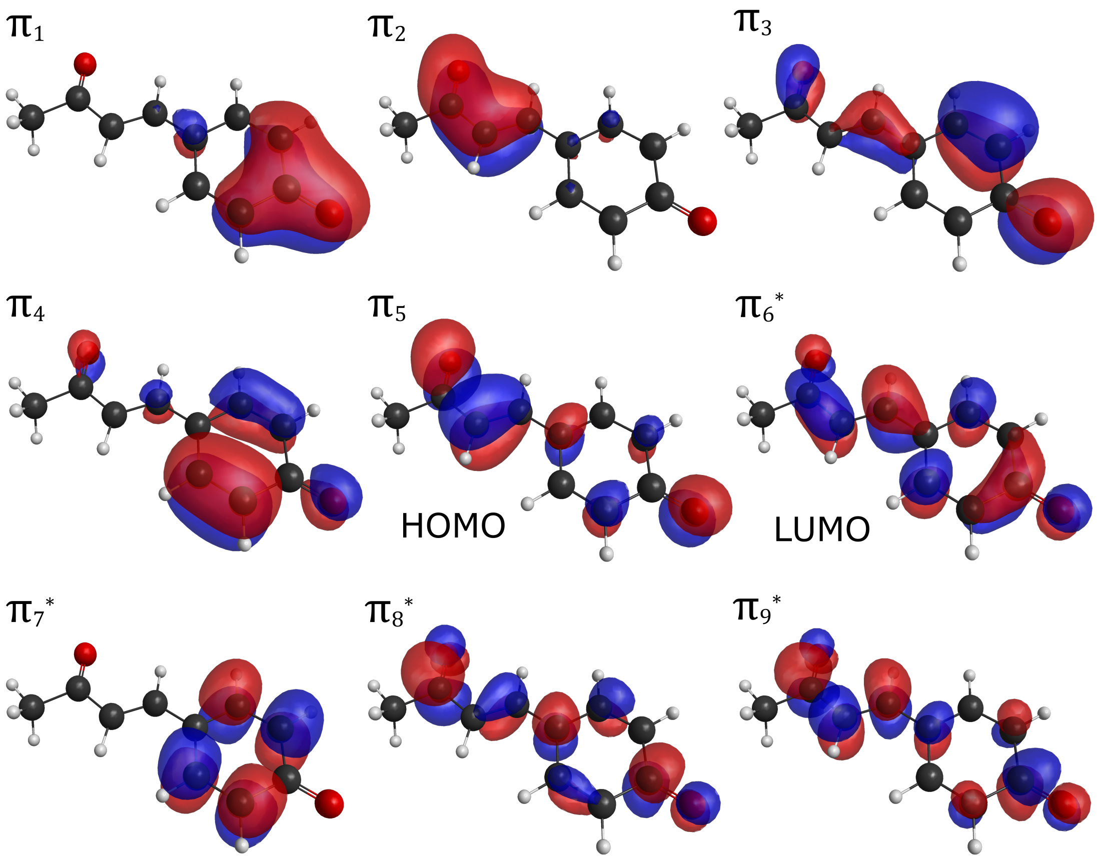

Figure S1: Optimized CASSCF orbitals for (E)-pCK^{-}.

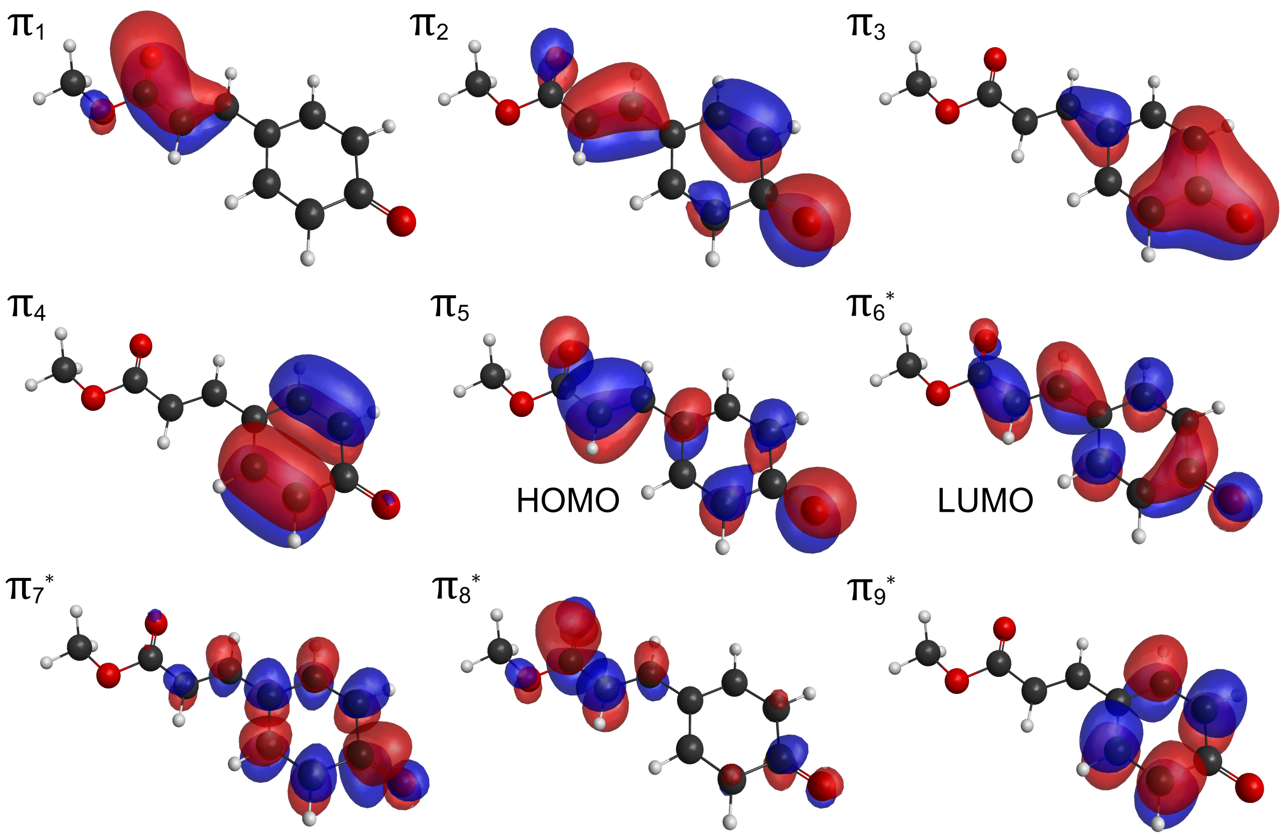

Figure S2: Optimized CASSCF orbitals for (E)-pCEs$^-$.

Action spectroscopy of pCK$^-$ in CO_2 buffer gas

In addition to the action spectra recorded in N_2 buffer gas (seeded with SF_6 and propan-2-ol) shown in the paper, action spectra were recorded in CO_2 buffer gas (Figure S3). The purpose of these measurements was two-fold: (i) to investigate if CO_2 buffer gas provided better separation of E and Z isomers compared with N_2 buffer gas,[22] and (ii) to investigate if the action spectra change appearance with buffer gas identity because CO_2 is more efficient at collisional energy transfer/quenching than N_2.

The photoaction ATDs for pCK$^-$ in pure CO_2 and CO_2 seeded with propan-2-ol are shown in Figure S3a and b, respectively. For pure CO_2 buffer gas, a small Z-isomer signal is observed on the

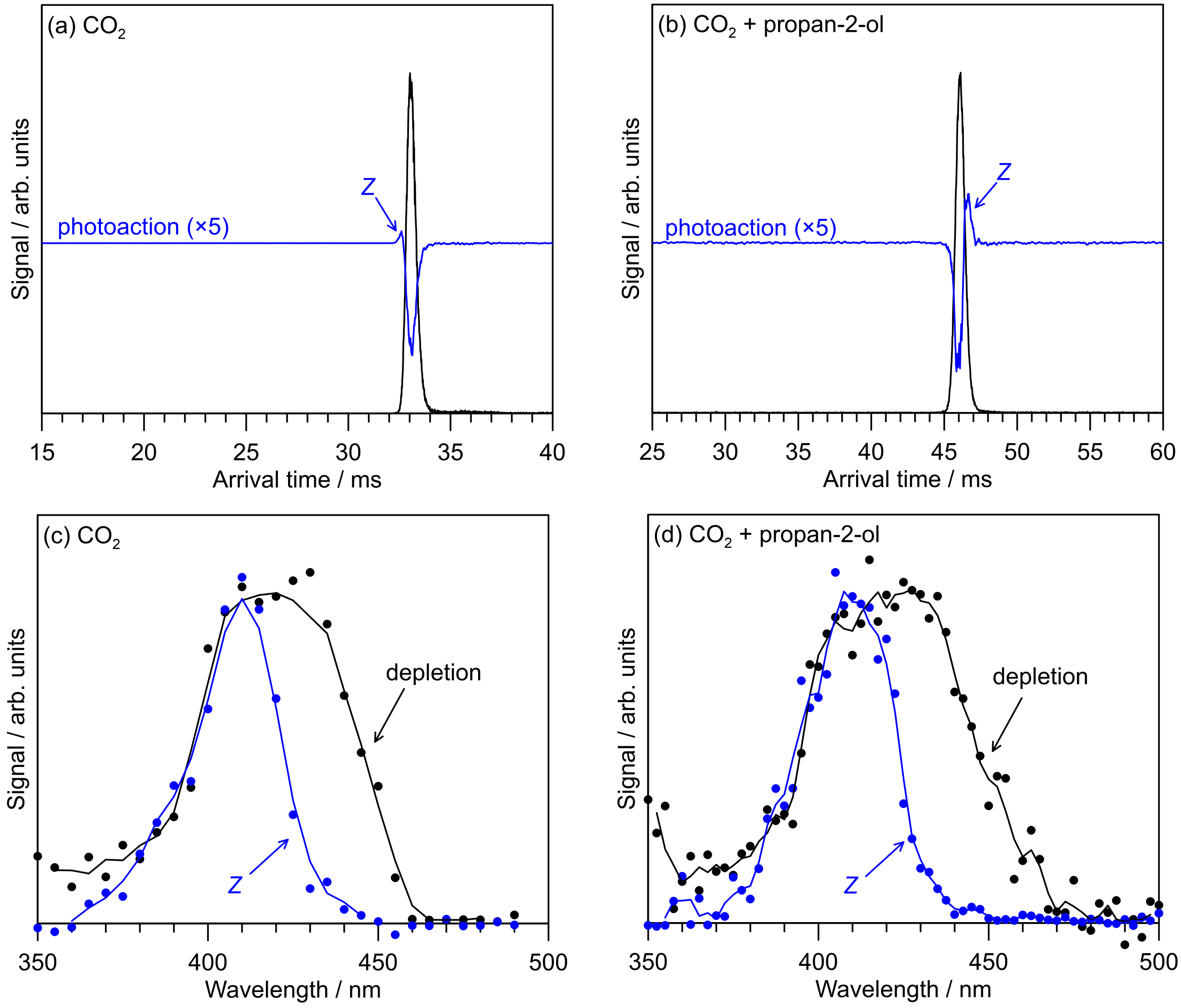

Figure S3: Ion mobility action spectroscopy of pCK$^-$: (a) light-off (black) and photoaction (blue) ATDs in CO_2 buffer gas, (b) light-off (black) and photoaction (blue) ATDs in CO_2 buffer gas seeded with ≈1% propan-2-ol, (c) action spectra recorded in CO_2 buffer gas and (d) action spectra recorded in CO_2 buffer gas seeded with ≈1% propan-2-ol. The photoaction spectra show the appearance (positive) and bleaching (negative) of signals. The quadrupole mass filter was set to the m/z of the parent ion.

fast side of the light-off ATD peak. For CO_2 seeded with ≈1% propan-2-ol, the *Z*-isomer signal is observed on the slower side of the light-off ATD peak and is slightly better resolved than in pure CO_2 buffer gas. Photodepletion and *E*-*Z* photoisomerization action spectra (normalized) are shown in Figure S3c and d, respectively. These sets of spectra closely resemble each other and parallel the action spectra reported in the paper, thus suggesting that that any differences in collisional energy quenching efficiency between N_2 and CO_2 buffer gas does not alter the appearance of the action spectra.

$\boldsymbol{p}$CK$^-$·propan-2-ol complexes

The addition of a small quantity of a dopant or 'mobility modifier' to the buffer gas used in ion mobility is a common practice to assist in separation of isomers with similar collision cross-sections.[23] Briefly, the collision cross-section, Ω, can be approximated as the sum of two contributions:

$$\Omega = \Omega_s + \Omega_l$$

where Ω_s is the small impact parameter term accounting for hard-sphere type interactions and Ω_l is the large impact parameter term accounting for glancing collisions. The latter of these terms is sensitive to long-range interactions (e.g. dipole-quadrupole and hydrogen bonding).

To help understand the larger collision cross-section for (Z)-pCK$^-$ compared with (E)-pCK$^-$ in N_2 + propan-2-ol (or CO_2 + propan-2-ol) buffer gas, we computed minimum energy structures and complex binding energies for the species shown in Figure S4. Based on DLPNO-CCSD(T)/aug-cc-pVDZ energies, the Z1-complex was $\approx$8 kJ mol^{-1} more stable (to dissociating) than the corresponding E1 complex, consistent with an increased Ω_l term. However, it is important to note that, because the photoisomerization and photodetachment (or photodepletion) spectra recorded in buffer gases with propan-2-ol dopant closely resemble those recorded in pure buffer gas, we conclude that any ion-molecule complexes are transitory and do no alter the action spectra.

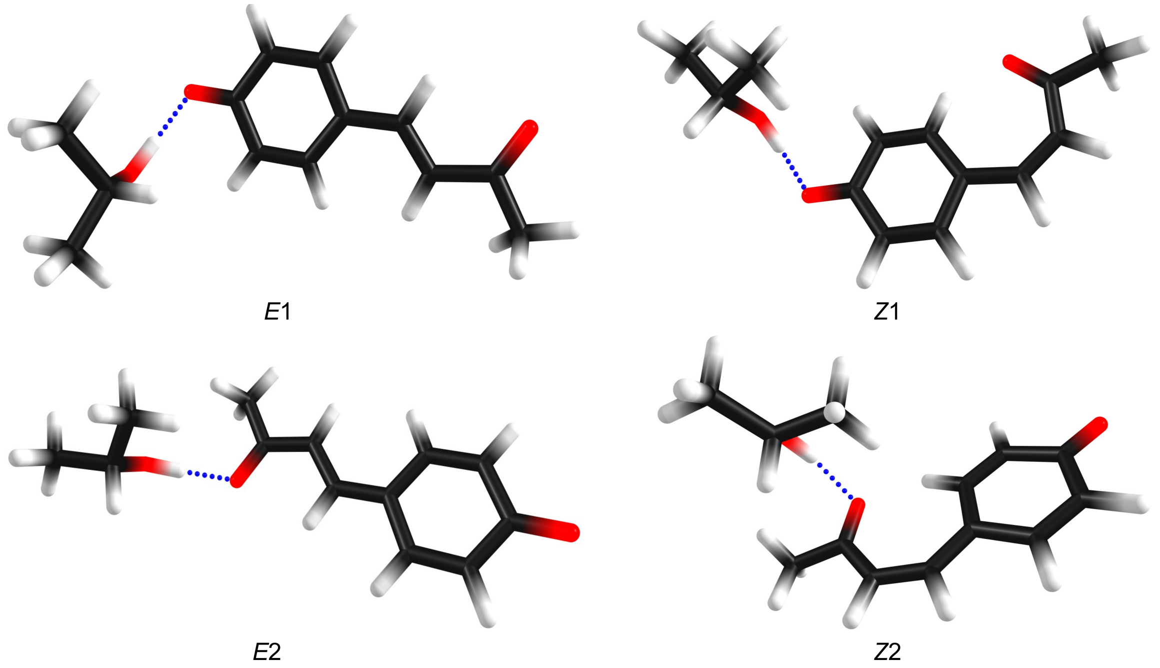

Figure S4: Calculated structures (ωB97X-D/aug-cc-pVDZ) for pCK$^-$·propan-2-ol complexes.

Solution spectroscopy

Sample preparation

Micromolar concentration solutions of pCK$^-$ were prepared using the following pure solvents: water (Milli-Q), methanol (99%, Alfa Aesar), ethanol (>99% Fisher), 1-propanol (Acros Organics), 2-propanol (>99.5%, Sigma-Aldrich), 1-butanol (>99%, Sigma-Aldrich), 1-pentanol (>99%, Sigma-Aldrich), 1-heptanol (99%, Lancaster), and ethylene glycol (>99%, Sigma-Aldrich). Further solutions were prepared using a mixture of water and ethylene glycol with ratios (vol% water): 80%, 60%, 40%, 20%. A drop of 1 M sodium hydroxide solution was used to deprotonate the neutral solutions and was sufficient to change the absorption spectrum fully (Figure S5). UV-vis absorption spectra were recorded using a Perkin-Elmer Lambda XLS spectrometer with a 1 cm path length quartz cuvette.

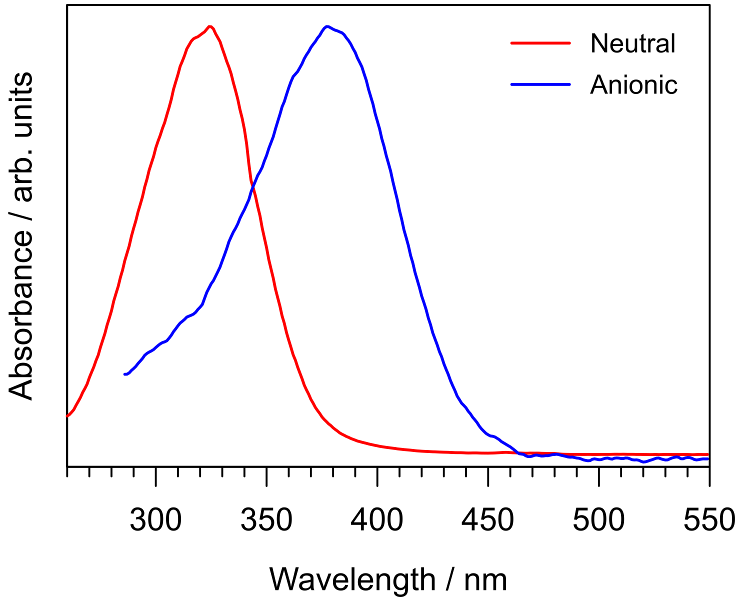

Figure S5: Absorption spectra for neutral (red, $\lambda_{max} = 324$ nm) and anionic (blue, $\lambda_{max} = 377$ nm) pCK in water at $T = 300$ K. The substantial spectral red shift with deprotonation is common to other *para*-hydroxycinnamates.[9]

Fluorescence spectra at 400 nm (pump wavelength for upconversion measurements) were recorded using a fluoroSENS fluorimeter (Gilden photonics) with a 1 cm path length quartz cuvette. Fluorescence excitation and emission spectra at $T = 77$ K (liquid nitrogen bath) were recorded using an Edinburgh Instruments FS5 spectrofluorometer and a Wilmad 5 mm diameter quartz EPR tube. Time-resolved fluorescence measurements at $T = 77$ K were initiated by pulses of light from an EPLED-360 LED source (365 nm, 400 ps FWHM instrument response function).

Solution spectroscopy

Fluorescence excitation and emission spectra for pCK$^-$ in ethanol at $T = 300$ K and $T = 77$ K are shown in Figure S6a. There is a substantial decrease in Stokes shift from 4143±20 cm^{-1} ($T = 300$ K) to 2243±20 cm^{-1} ($T = 77$ K). The large Stokes shift at $T = 300$ K is because of nuclear and solvent relaxation from the Franck-Condon geometry to the fluorescing geometry. In contrast, the same extent of nuclear reorganization does not occur in the glassy matrix at $T = 77$ K. Absorption and

fluorescence emission spectra for pCK$^-$ in water–ethylene-glycol mixtures are shown in Figure S7. Spectral properties of pCK$^-$ in the various solvents at $T = 300$ K are summarized in Table S1.

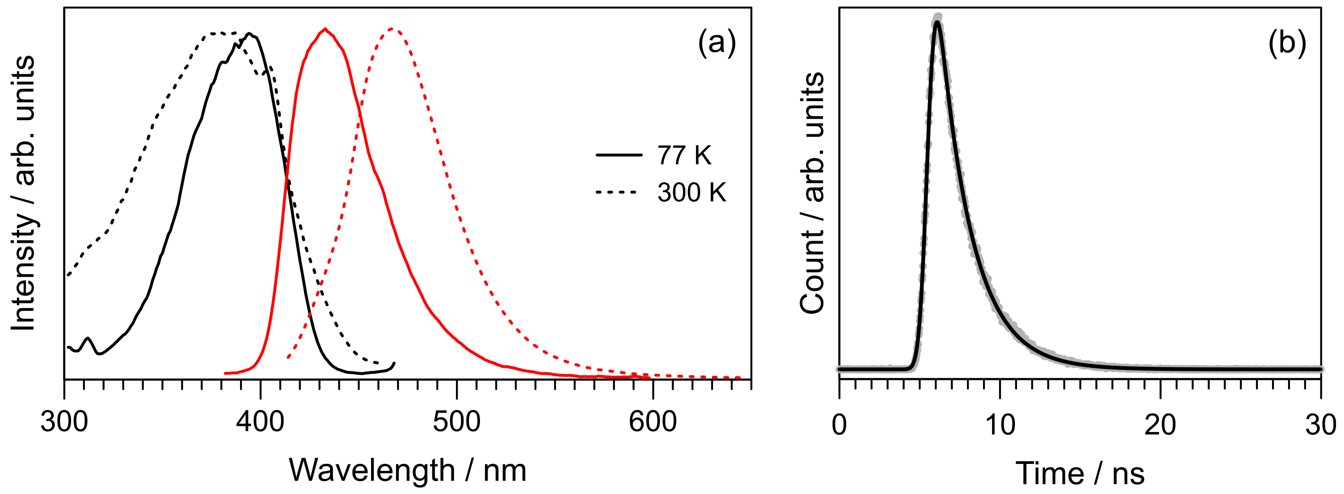

Figure S6: Fluorescence spectroscopy of pCK$^-$ in ethanol: (a) excitation and emission spectra at $T = 300$ K (dashed lines) and $T = 77$ K (solid lines). (b) time-resolved fluorescence data at $T = 77$ K exciting at 365 nm and monitoring fluorescence at 440 nm. Fit involves a Gaussian cross-correlation function convoluted with an exponential decay, giving the lifetime of $\tau = 2.04 \pm 0.03$ ns.

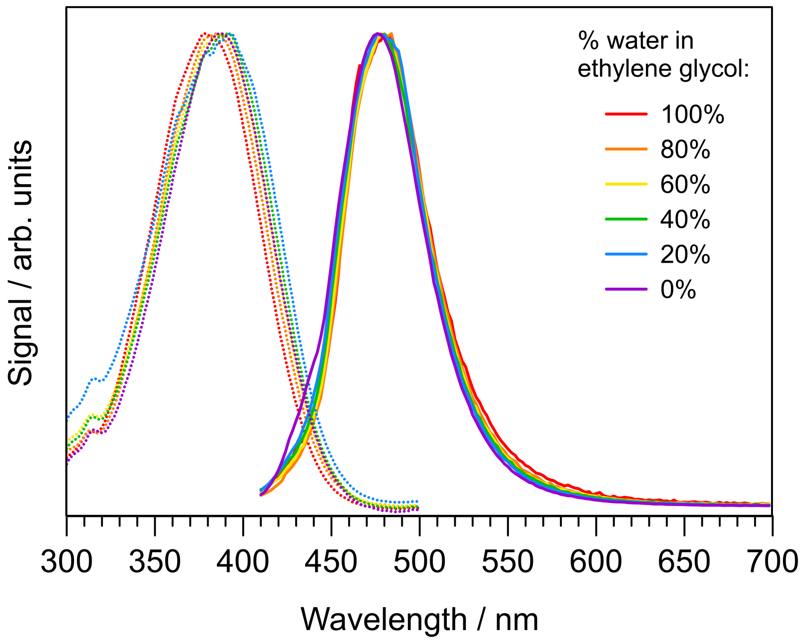

Figure S7: Absorption (dashed lines) and fluorescence emission (solid lines) species for pCK$^-$ in water–ethylene-glycol mixtures at $T = 300$ K.

Table S1: Absorption (λ_{max}^{A}, $\pm 1\,nm$) and emission (λ_{max}^{E}, $\pm 1\,nm$) maxima with corresponding Stokes shift $\Delta\nu$ ($\pm 20\,cm^{-1}$) for pCK^{-} at $T = 300\,K$. Percentages indicate vol% of water in the water–ethylene-glycol solutions. *Likely an outlier due to traces of water in the solvent.

Solvent	λ_{max}^{A} / nm	λ_{max}^{E} / nm	$\Delta\nu$ / cm^{-1}
H_2O	378	484	5794
80% H_2O	385	484	5313
60% H_2O	387	480	5006
40% H_2O	391	476	4567
20% H_2O	393	476	4437
EtGly	388	476	4765
MeOH	385	480	5141
EtOH	392	468	4143
1-PropOH	405	466	3232
2-PropOH	409	466	2991
ButOH*	394	468	4013
PentOH	400	468	3632
HeptOH	397	462	3544
OctOH	394	466	3921

Fluorescence upconversion

To explore the dependence of fluorescence lifetime on emission wavelength, up-converted signals from pCK$^-$ in water were monitored at a series of wavelengths (Figure S8). These data show the fluorescence lifetime to increase at longer monitoring wavelength. This is presumably because the lifetimes (close to 1 ps) are in competition with vibrational energy relaxation and nuclear reorganisation processes.[24] Following the discussion in Ref. 11, these lifetimes are comparable with the ≈880 fs long component of solvent dynamics in water.[25] For consistency in measurements across solvents, fluorescence lifetimes were recorded for up-converted signals corresponding to the wavelength of maximum response in the emission spectrum. Lifetimes extracted from fits to the experimental data are summarized in Table S2. When differences exist between static and flow cell fluorescence lifetimes, flow cell lifetimes are assumed more reliable due to establishment of a partial photostationary state in the static cell.

Fits to the upconversion data (e.g. see Figure S10) assumed either a Gaussian-like instrument response function convoluted with a single-exponential decay ($S_1 \xrightarrow{\tau_0} S_0$) or a double-exponential decay scheme $S_1 \xrightarrow{\tau_1} S_{1,rel} \xrightarrow{\tau_2} S_0$, where $S_{1,rel}$ is the relaxed excited state after solvent reorganization.[25] In most cases, τ_1 is limited by the cross correlation of the experiment. The lifetimes and fit residuals indicate that the double-exponential fit is most beneficial for the more viscous, less polar solvents due to slower solvent reorganization. These data improve on earlier upconversion experiments on pCK$^-$ with ≈500 fs time resolution.[11] For example, for pCK$^-$ in water, Espagne *et al.*[11] quoted $\tau_1 = 0.5$ ps and $\tau_2 = 1.3$ ps, to be compared with $\tau_1 = 0.055$ ps (limited by cross correlation) and $\tau_2 = 1.17$ ps (1.13 ps in a static cell) in the current work.

In water, there is only a 0.04 ps difference between fluorescence lifetimes measured in the flow and static cell, which although small, is beyond fitted uncertainty. This suggests that either there is only a small amount of Z-isomer formed, or the E and Z isomers have similar fluorescence lifetimes. The difference is more pronounced in viscous solvents.

Fluorescence lifetime trends for pCK$^-$ recorded water–ethylene-glycol mixtures are shown in

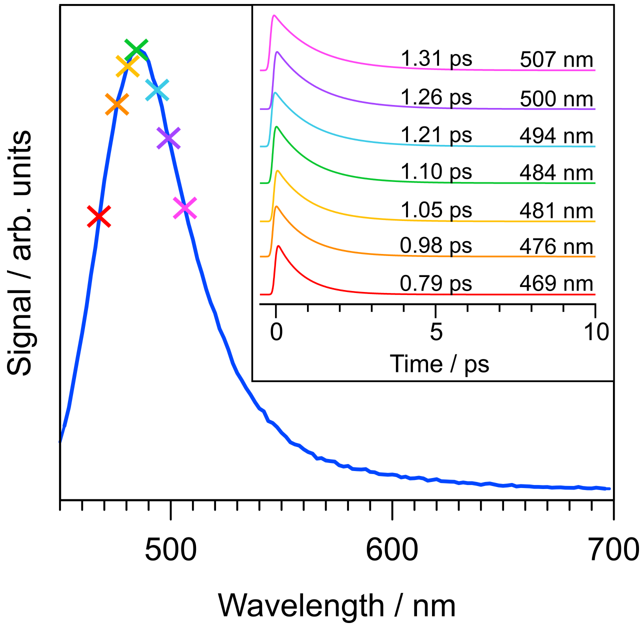

Figure S8: Wavelength dependence of observed fluorescence lifetime for pCK$^-$ in water excited at 400 nm (at $T = 300$ K). Wavelengths given in the inset are the monitoring positions over the emission band.

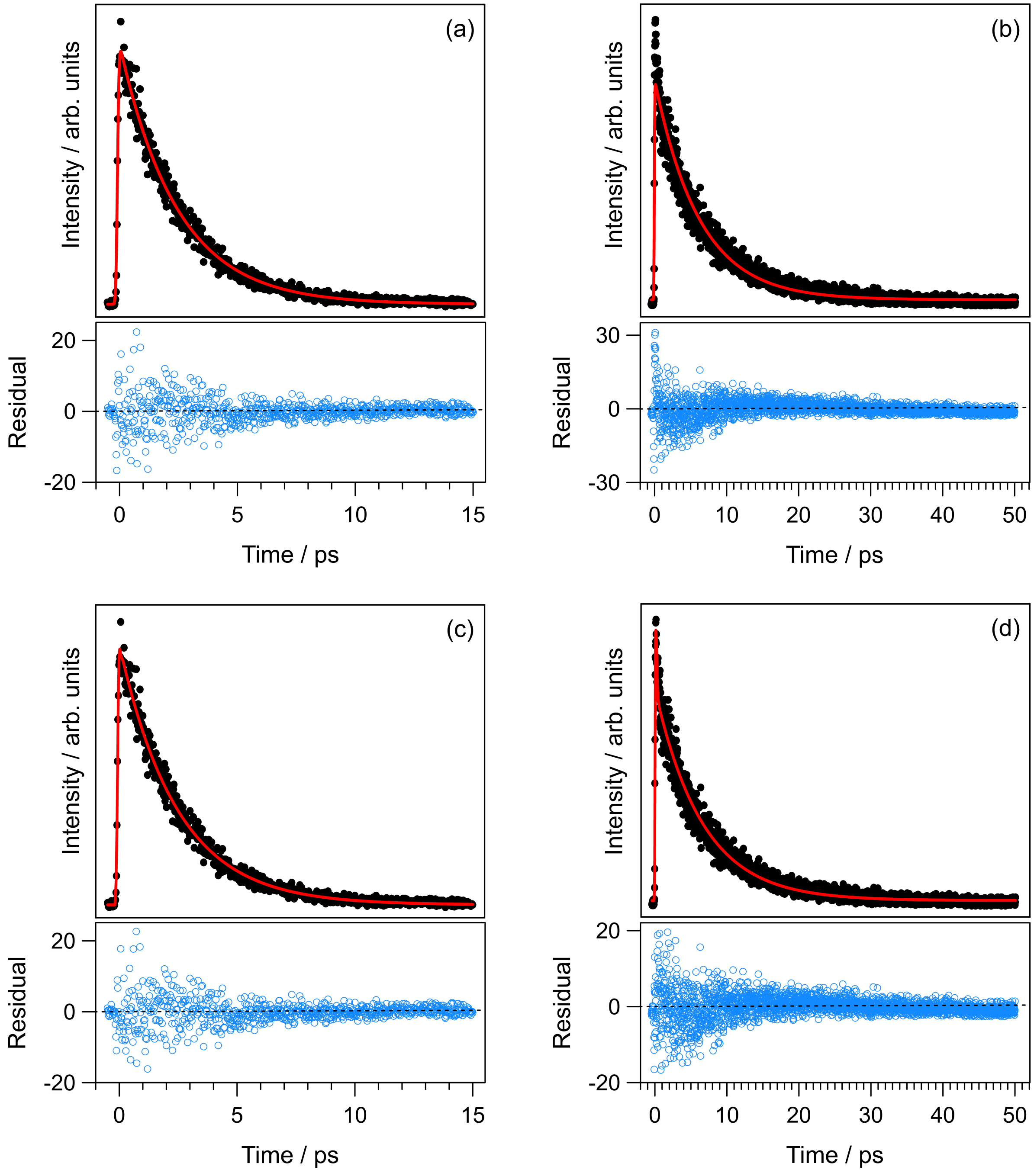

Figure S9: Example fluorescence upconversion data, fits and residuals for pCK$^-$ excited at 400 nm (at $T = 300$ K). (a) methanol single-exponential fit, (b) octanol single-exponential fit, (c) methanol double-exponential fit, and (d) octanol double-exponential fit.

Figure S10 (fitted lifetimes are tabulated in Table S2).

Dependence of the fitted excited state-lifetime for pCK$^-$ on the solvent polarity (dielectric constant) is summarized in Figure S11. Following an earlier study,[11] there are linear correlations between $\ln(k_f)$ and $\frac{1}{\varepsilon}$ for alcohol solvents (flow cell) and water–ethylene-glycol mixtures (static cell), and are related to the radii and charges or dipole moments of the reactant and excited state barrier.[26] However, quantitative information is difficult to extract because the viscosity dependence

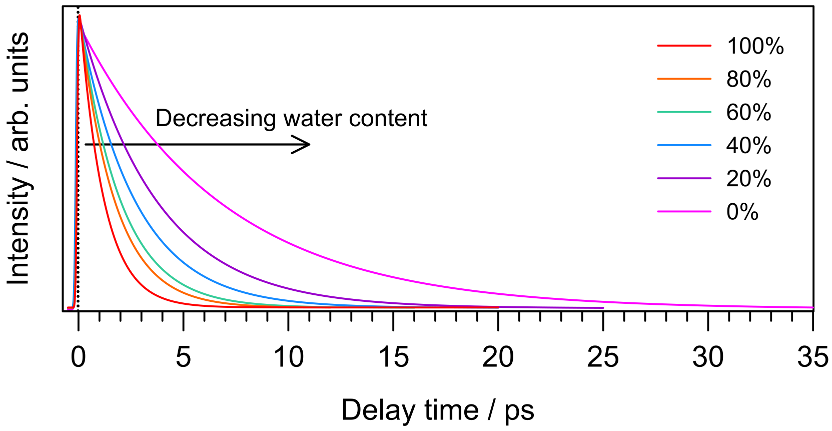

Figure S10: Fluorescence decay fits for pCK$^-$ in water–ethylene-glycol mixtures. Percentages indicate vol% of water at $T = 300\,\mathrm{K}$. Fitted lifetimes are given in Table S2.

is large. For the water–ethylene-glycol mixtures, it is reasonable to assume preferential solvation of the chromophore (e.g. first coordination sphere) by water molecules, leading to a degree of non-linearity in the correlation in Figure S11b. In the framework of electrostatic solute-solvent interactions, a negative slope in the correlations in Figure S11 show that excited state lifetime increases with solvent polarity and suggest that a more polar solvent decreases the barrier height by stabilizing the product state. This trend has been interpreted as consistent with a substantial charge shift accompanying excitation.[11,27,28]

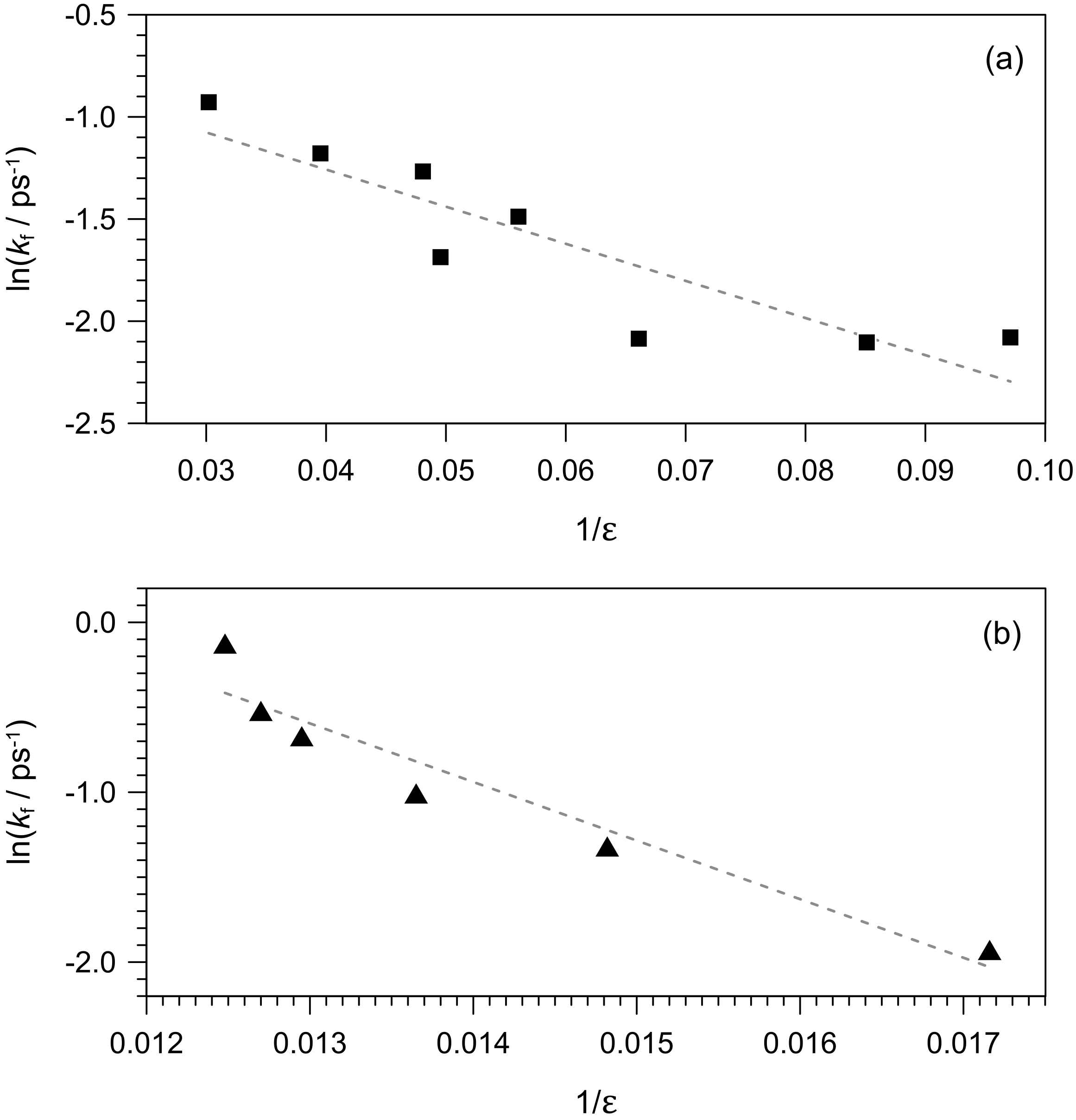

Figure S11: Dependence of $k_f \approx \frac{1}{\tau_2}$ for pCK$^-$ on solvent polarity, ϵ (dielectric constant), at $T =$ 300 K: (a) a series of alcohols using a flow cell, (b) water–ethylene-glycol mixtures using a static cell.

Table S2: Fluorescence lifetimes (in ps) of pCK$^-$ in various solvents and solvent mixtures measured using static (s) and flow (f) cells (at $T = 300\,$K), and obtained from single- (τ_0) and double-exponential fitting (τ_1 and τ_2). τ_1 is limited by experiment cross-correlation (55 fs). Percentages indicate vol% of water in the water–ethylene-glycol solutions. ± indicates the fitted uncertainty. EtGly is ethylene glycol.

	Single decay				Double decay							
**Solvent**	τ_0^s	±	τ_0^f	±	τ_1^s	±	τ_2^s	±	τ_1^f	±	τ_2^f	±
MeOH	2.53	0.01	2.44	0.02	0.055	-	2.53	0.01	0.055	-	2.45	0.02
EtOH	3.18	0.02	2.33	0.04	0.055	-	3.25	0.01	0.055	-	2.33	0.04
1-PropOH	3.43	0.07	3.31	0.03	0.055	-	3.55	0.08	0.055	-	3.40	0.07
2-PropOH	5.27	0.05	3.70	0.04	0.055	-	5.40	0.06	0.055	-	3.84	0.04
ButOH	4.31	0.02	5.32	0.05	0.055	-	4.43	0.01	0.055	-	5.47	0.05
PentOH	7.71	0.04	6.96	0.08	0.069	0.002	8.05	0.04	0.055	-	7.11	0.08
HeptOH	7.82	0.06	8.01	0.11	0.080	0.002	8.20	0.06	0.091	0.008	8.44	0.10
OctOH	7.67	0.05	6.19	0.04	0.055	-	8.00	0.05	0.080	0.003	6.57	0.04
H_2O	1.14	0.04	1.18	0.01	0.055	-	1.13	0.00	0.055	-	1.17	0.01
EtGly	6.86	0.04	5.60	0.12	0.080	0.001	6.86	0.04	0.055	-	5.44	0.04
80% H_2O	1.67	0.01	-	-	0.055	-	1.68	0.01	-	-	-	-
60% H_2O	1.95	0.01	-	-	0.055	-	1.95	0.01	-	-	-	-
40% H_2O	2.68	0.02	-	-	0.055	-	2.73	0.02	-	-	-	-
20% H_2O	3.69	0.04	-	-	0.055	-	3.73	0.02	-	-	-	-

References

(1) Campbell, J. L.; Zhu, M.; Hopkins, W. S. Ion-Molecule Clustering in Differential Mobility Spectrometry: Lessons Learned From Tetraalkylammonium Cations and Their Isomers. *J. Am. Soc. Mass Spectrom.* **2014**, *25*, 1583–1591.

(2) Liu, C.; Le Blanc, Y.; Shields, J.; Janiszewki, J.; Ieritano, C.; Ye, G.; Hawes, G.; Hopkins, S.; Campbell, J. L. Using Differential Mobility Spectrometry to Measure Ion Solvation: An Examination of the Roles of Solvents and Ionic Structures in Separating Quinoline-Based Drugs. *Analyst* **2015**, *140*, 6897–6903.

(3) Liu, C.; Le Blanc, J. C. Y.; Schneider, B. B.; Shields, J.; Federico, J. J.; Zhang, H.; Stroh, J. G.; Kauffman, G. W.; Kung, D. W.; Ieritano, C. et al. Assessing Physicochemical Properties of Drug Molecules via Microsolvation Measurements With Differential Mobility Spectrometry. *ACS Cent. Sci.* **2017**, *3*, 101–109.

(4) Coughlan, N. J.; Carr, P. J.; Walker, S. C.; Zhou, C.; Guna, M.; Campbell, J. L.; Hopkins, W. S. Measuring Electronic Spectra of Differential Mobility-Selected Ions in the Gas Phase. *J. Am. Soc. Mass Spectrom.* **2020**, *31*, 405–410.

(5) Londry, F. A.; Hager, J. W. Mass Selective Axial Ion Ejection From a Linear Quadrupole Ion Trap. *J. Am. Soc. Mass Spectrom.* **2003**, *14*, 1130–1147.

(6) Adamson, B. D.; Coughlan, N. J. A.; Continetti, R. E.; Bieske, E. J. Changing the Shape of Molecular Ions: Photoisomerization Action Spectroscopy in the Gas Phase. *Phys. Chem. Chem. Phys.* **2013**, *15*, 9540–9548.

(7) Adamson, B. D.; Coughlan, N. J. A.; Markworth, P. B.; Continetti, R. E.; Bieske, E. J. An Ion Mobility Mass Spectrometer for Investigating Photoisomerization and Photodissociation of Molecular Ions. *Rev. Sci. Instr.* **2014**, *85*, 123109.

(8) Eiceman, G. A.; Karpas, Z.; Hill, H. H. *Ion Mobility Spectrometry*, 3rd ed.; CRC Press, 2013.

(9) Bull, J. N.; da Silva, G.; Scholz, M. S.; Carrascosa, E.; Bieske, E. J. Photoinduced Intramolecular Proton Transfer in Deprotonated *para*-Coumaric Acid. *J. Phys. Chem. A* **2018**, *123*, 4419–4420.

(10) Heisler, I. A.; Kondo, M.; Meech, S. R. Reactive Dynamics in Confined Liquids: Ultrafast Torsional Dynamics of Auramine O in Nanoconfined Water in Aerosol OT Reverse Micelles. *J. Phys. Chem. B* **2009**, *113*, 1623–1631.

(11) Espagne, A.; Paik, D. H.; Changenet-Barret, P.; Martin, M. M.; Zewail, A. H. Ultrafast Photoisomerization of Photoactive Yellow Protein Chromophore Analogues in Solution: Influence of the Protonation State. *ChemPhysChem* **2006**, *7*, 1717–1726.

(12) Frisch, M. J.; Trucks, G. W.; Schlegel, H. B.; Scuseria, G. E.; Robb, M. A.; Cheeseman, J. R.; Scalmani, G.; Barone, V.; Mennucci, B.; Petersson, G. A. et al. Gaussian 16 Revision B.01. Gaussian Inc. Wallingford CT 2016.

(13) Granovsky, A. A. Firefly Version 8.2.0. http://classic.chem.msu.su/gran/firefly/index.html.

(14) Neese, F. The ORCA Program System. *WIRES Comp. Mol. Sci.* **2012**, *2*, 73–78.

(15) Chai, J.-D.; Head-Gordon, M. Long-Range Corrected Hybrid Density Functionals with Damped Atom-Atom Dispersion Corrections. *Phys. Chem. Chem. Phys.* **2008**, *10*, 6615–6620.

(16) Dunning, T.H., Jr., Gaussian Basis Sets for Use in Correlated Molecular Calculations. I. The Atoms Boron Through Neon and Hydrogen. *J. Chem. Phys.* **1989**, *90*, 1007.

(17) Riplinger, C.; Sandhoefer, B.; Hansen, A.; Neese, F. Natural Triple Excitations in Local Coupled Cluster Calculations With Pair Natural Orbitals. *J. Chem. Phys.* **2013**, *139*, 134101.

(18) Campuzano, I.; Bush, M. F.; Robinson, C. V.; Beaumont, C.; Richardson, K.; Kim, H.; Kim, H. I. Structural Characterization of Drug-Like Compounds by Ion Mobility Mass Spectrometry: Comparison of Theoretical and Experimentally Derived Nitrogen Collision Cross Sections. *Anal. Chem.* **2012**, *84*, 1026–1033.

(19) Mesleh, M. F.; Hunter, J. M.; Shvartsburg, A. A.; Schatz, G. C.; Jarrold, M. F. Structural Information From Ion Mobility Measurements: Effects of the Long-Range Potential. *J. Phys. Chem.* **1996**, *100*, 16082–16086.

(20) Besler, B. H.; Merz, Jr., K. M.; Kollman, P. A. Atomic Charges Derived From Semiempirical Methods. *J. Comp. Chem.* **1990**, *11*, 431–439.

(21) Boggio-Pasqua, M.; Groenhof, G. On the Use of Reduced Active Space in CASSCF Calculations. *Comput. Theo. Chem.* **2014**, *1040-1041*, 6–13.

(22) Bull, J. N.; Coughlan, N. J. A.; Bieske, E. J. Protomer-Specific Photochemistry Investigated Using Ion Mobility Mass Spectrometry. *J. Phys. Chem. A* **2017**, *121*, 6021–6027.

(23) Waraksa, E.; Perycz, U.; Namieśnik, J.; Sillanpää, M.; Dymerski, T.; Wójtowicz, M.; Puton, J. Dopants and Gas Modifiers in Ion Mobility Spectrometry. *Trends Anal. Chem.* **2016**, *82*, 237–249.

(24) Tramer, A.; Jungen, C.; Lahmani, F. *Energy Dissipation in Molecular Systems*; Springer London, Limited, 2005.

(25) Jimenez, R.; Fleming, G. R.; Kumar, P. V.; Maroncelli, M. Femtosecond Solvation Dynamics of Water. *Nature* **1994**, *369*, 471–473.

(26) Reichardt, C.; Welton, T. *Solvents and Solvent Effects in Organic Chemistry*; Wiley-VCH Verlag GmbH & Co. KGaA, 2010.

(27) Espagne, A.; Paik, D. H.; Changenet-Barret, P.; Plaza, P.; Martin, M. M.; Zewail, A. H. Ultrafast Light-Induced Response of Photoactive Yellow Protein Chromophore Analogues. *Photochem. Photobiol. Sci.* **2007**, *6*, 780–787.

(28) Espagne, A.; Changenet-Barret, P.; Baudin, J.-B.; Plaza, P.; Martin, M. M. Photoinduced Charge Shift as the Driving Force For the Excited-State Relaxation of Analogues of the Photoactive Yellow Protein Chromophore in Solution. *J. Photochem. Photobiol. A: Chem.* **2007**, *185*, 245–252.
